# Oxidative Stress, Genetic Factors and Behavioral Responses to Chemical Exposure: Insights into Cancer Development in Zebrafish (Danio Rerio)

**DOI:** 10.1007/s10528-025-11148-6

**Published:** 2025-06-03

**Authors:** Cătălina Ionescu, Viorica Rarinca, Mălina Visternicu, Alin Ciobica, Laura Romila, Vasile Burlui, Mirela Cimpeanu, Bogdan Novac, Bogdan Gurzu

**Affiliations:** 1 Department of Biology, Faculty of Biology, Alexandru Ioan Cuza University of Iasi, Bd. Carol I No. 20A, 700505 Iasi, Romania; 2 “Ioan Haulica” Institute, Apollonia University, Pacurari Street 11, 700511 Iasi, Romania; 3 Doctoral School of Biology, Faculty of Biology, Alexandru Ioan Cuza University of Iasi, No 20A, Carol I Avenue, 700506 Iasi, Romania; 4 Faculty of Geography and Geology, “Alexandru Ioan Cuza”, Doctoral School of Geosciences, University of Iași, Carol I Avenue, No. 20A, 700505 Iași, Romania; 5 CENEMED Platform for Interdisciplinary Research, University of Medicine and Pharmacy “Grigore T. Popa”, 700115 Iasi, Romania; 6 Academy of Romanian Scientists, No 54, Independence Street, Sector 5, 050094 Bucharest, Romania; 7 Clinical Department, Apollonia University, Păcurari Street 11, 700511 Iasi, Romania; 8 Faculty of Medicine, University of Medicine and Pharmacy “Grigore T. Popa”, 700115 Iasi, Romania

**Keywords:** Zebrafish, Cancer, Chemicals, Oxidative stress, Genetic factors, Carcinogenic, Behavior

## Abstract

Cancer research in animal models is critical for understanding disease mechanisms and testing potential therapies. Among the various models, zebrafish (Danio rerio) has gained prominence due to its genetic similarity to humans, rapid development, and transparency during early stages. The objective of this article is to explore the zebrafish as a powerful model for studying cancer, particularly focusing on the role of oxidative stress (OS) and genetic factors (oncogenes, tumor suppressor genes) in cancer progression. The interaction between oxidative stress, DNA damage, and genetic mutations is examined, highlighting how these processes contribute to tumor formation. Additionally, the study investigates the various behavioral tests used to assess the effects of carcinogenic and non-carcinogenic substances, such as sodium arsenate (As(V), N-ethyl-N-nitrosourea (ENU), dimethylbenzanthracene (DMBA), N-methyl-N1-nitro-N-nitrosoguanidine (MNNG), Atrazine and Methylmercury (MeHg) on zebrafish cognitive functions. The review included 170 papers published in English up to December 2024 and was conducted in accordance with PRISMA guidelines, using multiple electronic databases (Science Direct, PubMed and Google Scholar) to perform a comprehensive and systematic search using keywords. Through this approach, zebrafish emerge as a versatile model for cancer research, offering insights into both the physiological and behavioral impacts of chemical exposures.

**Graphic Abstract:**

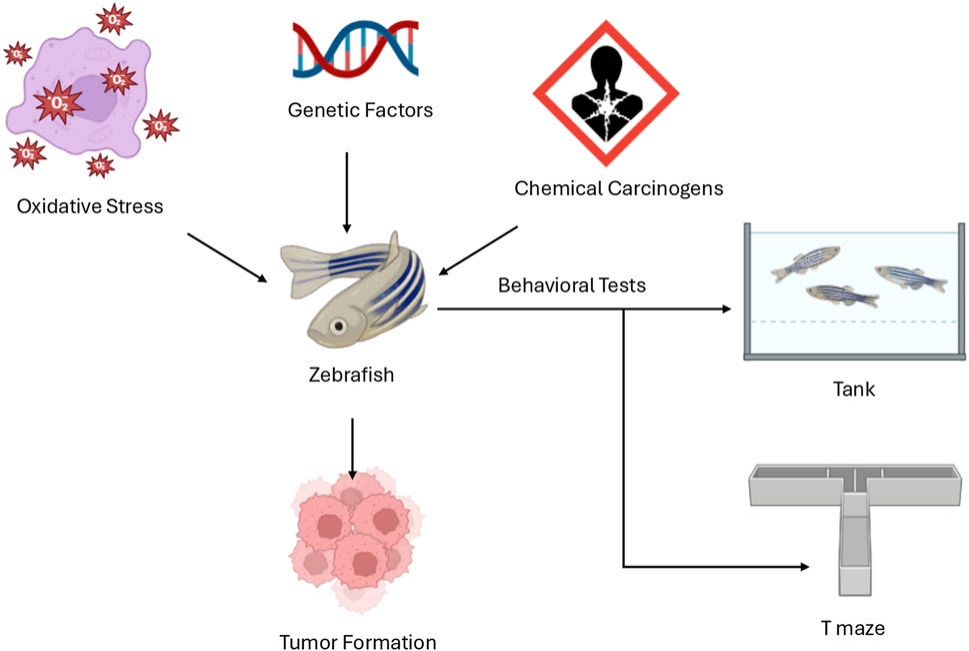

## Introduction

Chemical exposure plays a critical role in cancer progression by initiating and promoting cellular alterations that contribute to tumor formation (Casey et al. 2015). Several environmental and industrial chemicals have been identified as carcinogens, including As(V), a toxic metalloid known to induce oxidative stress and DNA damage (Xu et al. 2013); ENU, a potent alkylating agent that causes mutations leading to tumorigenesis (Solnica-Krezel et al. 1994); DMBA, a polycyclic aromatic hydrocarbon that triggers DNA adduct formation and genetic instability (Spitsbergen et al. 2000); and MNNG, a direct-acting mutagen that interferes with DNA repair mechanisms (Shen et al. 2023). Additionally, atrazine, a widely used herbicide, has been implicated in endocrine disruption and carcinogenic effects (Wirbisky et al. 2016), while MeHg, a highly toxic organic mercury compound, contributes to cellular toxicity and epigenetic modifications (Carvan et al. 2017). Specific chemicals, including carcinogens can cause DNA damage, disrupt cellular processes, and induce genetic mutations, all of which contribute to cancer development (Casey et al. 2015). These chemicals induce DNA damage through several mechanisms, such as the formation of single-strand breaks, cross-linking of DNA strands, and the generation of DNA adducts (Zhang et al. 2022). Single-strand breaks (SSBs) happen when one of the two DNA strands in the double helix is broken. These breaks can be caused by factors such as oxidative stress, radiation, or chemical exposure. Although SSBs are less damaging than double-strand breaks, they still present a considerable risk if not repaired, as they can cause errors during DNA replication and lead to genomic instability. If left unrepaired, SSBs may result in mutations and contribute to the onset of cancer (Caldecott 2008). For instance, O^6^-alkylguanine lesions can be indirectly connected to SSBs through the cellular response to DNA damage, particularly in the context of mismatch repair (MMR). ENU induces O⁶-alkylguanine lesions in DNA, which are recognized by the MMR system but are not repairable. This recognition leads to replication fork stalling and subsequent chromosomal instability. Notably, zebrafish embryos deficient in the MMR component MSH6 exhibited reduced lethality and mutation frequencies upon ENU exposure compared to wild-type embryos, underscoring the role of MMR in mediating the cytotoxic effects of ENU-induced DNA damage (Feitsma et al. 2008).

DNA cross-linking occurs when covalent bonds are formed between the two DNA strands or between DNA and proteins, hindering the proper separation of the strands during replication and transcription. This cross-linking can be triggered by chemotherapy drugs or environmental chemicals. If these cross-links are not repaired, they can cause cell death or contribute to cancer development by inducing chromosomal instability (Legerski 2010). DNA adducts are created when chemical carcinogens bind covalently to DNA bases, leading to distortions in the DNA double helix. These distortions disrupt normal base pairing during DNA replication and transcription. Carcinogens, such as benzo[a]pyrene, are known to form bulky adducts that cause mutations. If these adducts are left unrepaired, they can result in lasting genetic damage, ultimately promoting the development of cancer (Zhang et al. 2022). Although ENU is a monofunctional alkylating agent and does not induce DNA cross-links, several studies have demonstrated that zebrafish respond to cross-linking agents through well-conserved DNA damage pathways. For instance, exposure to 4,5′,8-trimethylpsoralen (TMP) has been shown to cause mutagenic effects in zebrafish embryos, with phenotypes linked to neural development defects (Ando and Mishina 1998). Similarly, diepoxybutane (DEB), a bifunctional cross-linking agent, induces thrombosis defects in zebrafish larvae due to vascular injury (Raman et al. 2022).

As discussed before, alkylating agents like ENU can cause guanine alkylation, leading to mismatched base pairing during DNA replication (Hoyos-Manchado et al. 2020). Similarly, polycyclic aromatic hydrocarbons like DMBA can form DNA adducts that disrupt normal base pairing and lead to mutations (Manjanatha et al. 2000). These alterations can compromise genomic integrity, impair DNA repair mechanisms, and drive tumorigenesis through the accumulation of mutations and chromosomal instability. Alongside chemical exposure, OS is a key factor, as reactive oxygen species (ROS) generated during cellular metabolism or in response to environmental stress can further damage DNA, proteins, and lipids, accelerating carcinogenesis (Hong et al. 2024). One significant type of oxidative DNA damage is the formation of 8-hydroxy-2′-deoxyguanosine (8-OHdG), a biomarker of oxidative stress. 8-OHdG results from the modification of guanine and, if not properly repaired, can lead to mutations, such as G·C to T·A transversions, during DNA replication. These mutations contribute to genomic instability, a hallmark of carcinogenesis. Elevated levels of 8-OHdG are associated with an increased risk of various cancers, making it an important indicator of oxidative damage in cancer development (Pande et al. 2015; Qing et al. 2019). Notably, 8-OHdG has also been quantified in zebrafish following carcinogen exposure using high-performance liquid chromatography (HPLC) and immunohistochemistry, highlighting its utility in modeling oxidative DNA damage in vivo (Gentile et al. 2023; Paola et al. 2021). Genetic factors also interact with these external insults, with mutations in tumor suppressor genes and oncogenes altering normal cellular functions, leading to uncontrolled cell growth. Together, chemical exposure, oxidative stress, and genetic factors create a complex environment that fosters cancer initiation and progression (Reuter et al. 2010).

Cancer is a genetically intricate disease characterized by the gradual accumulation of somatic mutations, along with occasional inherited mutations, which ultimately lead to the transformation of normal cells into clonal neoplastic cells. This multistep process highlights the complex interplay between genetic alterations and cellular mechanisms involved in tumor development (Berghmans et al. 2005a). Inherited mutations play a crucial role in several hereditary cancers, such as familial breast cancer (BRCA1/BRCA2 mutations), familial colon cancer (e.g., mutations in the APC gene), and retinoblastoma (mutations in the RB1 gene). These inherited mutations predispose individuals to cancer and can be modeled in zebrafish, providing valuable insights into cancer initiation and progression. For example, zebrafish models have been used to study BRCA1/BRCA2 mutations in breast cancer (Miki et al. 1979), APC mutations in colorectal cancer (Haramis et al. 2006), and RB1 mutations in retinoblastoma (Maricic et al. 2022), allowing researchers to observe tumor formation and test potential therapeutic approaches in a living organism.

To reduce the global prevalence of cancer, it is essential to investigate and advance innovative treatment options alongside new diagnostic techniques. These developments play a significant role in enhancing our capacity for early detection and effective interventions (Li et al. 2021a). Continued focus on this area is vital for improving cancer outcomes and overall public health. By enhancing our understanding of cancer biology and developing effective interventions, we can significantly improve early detection and therapeutic outcomes. The use of animals in scientific research is a topic that often brings about strong opinions. However, animal models play a pivotal role in our quest to understand complex biological and pathological processes. They are essential for the advancement of biomedical science, helping us to unlock the mysteries of diseases and refine treatment methods. These models are carefully chosen based on their functional and genetic characteristics, which align with specific research goals (Domínguez-Oliva et al. 2023).

In cancer research, animal models are invaluable for helping us grasp the genetic factors that contribute to cancer. They allow scientists to investigate how specific genes, and their mutations can lead to the onset and progression of the disease. By studying these models, researchers gain insights into the complex interplay between genetics and cancer development, which is essential for identifying potential treatments and preventative measures (Li et al. 2021a).

Zebrafish (Danio rerio), commonly known as zebrafish, have gained significant popularity as a model organism in research, particularly for studying developmental processes and human disorders (Zhao et al. 2015). This animal offers several unique advantages that make it an incredibly versatile tool in scientific research, especially for investigating human cancer biology and metastasis (Letrado et al. 2018; Astell and Sieger 2020). Its embryos are regarded as a valuable model for studying environmental exposures relevant to human health (Boix et al. 2020).

However, while zebrafish models provide numerous advantages, they also face certain limitations, particularly in replicating the intricate tumor microenvironment (TME) observed in humans. Similarly, traditional in vitro preclinical models, such as monolayer cell cultures, lack the complexity needed to mimic cancer biology fully. To overcome these limitations, advanced systems like 3D cultures, organ-on-chip platforms, and tissue cultures have emerged as valuable tools. Integrating zebrafish models with such systems can enhance the biological relevance and translational potential of research findings, bridging the gap between in vitro simplicity and in vivo complexity.

These approaches offer a pathway to improve the reliability of cancer studies, addressing the challenges posed by the limitations of individual models. Such integrative methodologies are crucial for a deeper understanding of tumor biology and for the development of effective therapeutic strategies.

## Methodology

### Search Strategy

The current systematic review was conducted following the Preferred Reporting Items for Systematic Review and Meta-Analysis (PRISMA) guidelines, employing several electronic databases (Science Direct, PubMed, and Google Scholar) to conduct a comprehensive and systematic search using the keywords. The inclusion criteria focused on studies published up to December 2024 in English, which evaluated the chemical agents exposure on cancer development in zebrafish. Also, the genetic factors involved were discussed, as well as the relevance of oxidative stress.

### Excluding Criteria

**Fig. 1:**
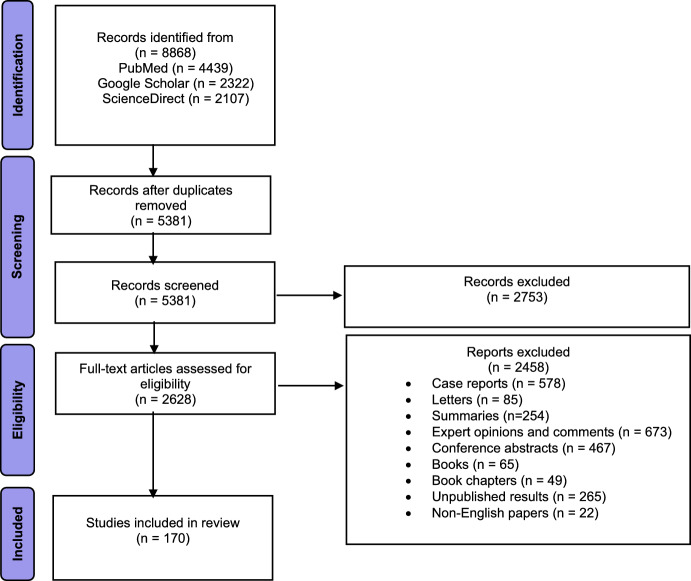
PRISMA flow chart illustrating the studies selection and exclusion criteria

We applied the following exclusion criteria, illustrated in Fig. 1.

### Data Extraction

Out of the initial 8868 reports collected through electronic search, 5381 were omitted due to duplication, 2753 were excluded based on article type, and an additional 2458 were excluded as they comprised conference abstracts, books, book chapters, unpublished result and excluded because they were not in English.

### Data Synthesis

Finally, 170 articles were included in this study, as shown in the diagram of the literature search and selection process (Fig. 1). Because of the variability among the studies, narrative synthesis was deemed the most suitable approach. The results are summarized in 4 chapters, where we highlighted relevant information about the role of oxidative stress, genetic factors and behavioral responses in cancer development using the zebrafish model, exposed to various chemical agents.

## Oxidative Stress—Mechanisms and Its Role in Cancer Development

### Definition, Basic Concepts of Oxidative Stress and Sources of ROS

Ranging from vertebrates to invertebrates, including rodents, fish, and both in vivo and in vitro systems, oxidative stress has been explored across a wide variety of model organisms. However, zebrafish—whether at the adult, larval, or embryonic stage—have emerged as one of the most promising vertebrate models for studying oxidative stress, due to several key advantages (Chowdhury and Saikia 2022).

Oxidative stress occurs when there is an imbalance between oxidants and antioxidants in the body, often due to an overproduction of ROS or a malfunctioning antioxidant system. This imbalance can lead to oxidative damage within cells, causing injury and even cell death, with ROS being a key contributor to this damage. The accumulation of pro-oxidant compounds plays a significant role in the development of various pathological conditions (Issac et al. 2021), such as atherosclerosis, chronic obstructive pulmonary disease, Alzheimer’s disease, and cancer (Forman and Zhang 2021). Zebrafish have a range of antioxidant systems, including enzymatic antioxidants such as superoxide dismutase (SOD), catalase (CAT), and glutathione peroxidase (GPx), as well as non-enzymatic antioxidants like glutathione (GSH). These systems work synergistically to neutralize the damaging effects of ROS. The expression of antioxidant enzymes in zebrafish varies throughout their development. In the early stages, embryos primarily rely on antioxidants provided by the mother. As the larvae grow, they begin to produce their own antioxidant enzymes, with increased levels of SOD and CAT activity, which enhance their capacity to mitigate oxidative stress. This regulation of antioxidant defense across developmental stages is essential for protecting zebrafish from oxidative damage that could lead to diseases, including cancer (Abbate et al. 2021; Li et al. 2022; Xu et al. 2022). This highlights the diverse mechanisms through which oxidants contribute to cellular damage in these conditions (Forman and Zhang 2021).

The effects of oxidative stress can vary widely depending on several factors, including the type of reactive species involved, the subcellular structures where they are produced, the specific organs or tissues affected, the organism’s genetic traits, and its developmental stage. OS has been linked to numerous processes such as aging, cancer, diabetes, and cardiovascular and neurodegenerative diseases (Boix et al. 2020). Fish possess homeostatic enzyme systems similar to those found in many mammals. During respiration, they produce ROS, which can interact with organic molecules. ROS can also be generated by intracellular enzymes such as nicotinamide adenine dinucleotide phosphate (NADPH) oxidase and cytoplasmic xanthine. When ROS levels exceed the cell’s buffering capacity, oxidative stress occurs, potentially leading to damage of DNA, RNA, proteins, and lipids (Hagedorn et al. 2012). The enzymes are essential for maintaining cellular function and responding to environmental stressors, helping to detoxify ROS, repair DNA damage, and maintain metabolic balance, thus preventing oxidative damage that could contribute to diseases such as cancer (Abbate et al. 2021).For instance, zebrafish express SOD, both Cu/Zn and Mn forms, which neutralize superoxide radicals, a major source of ROS. By converting these radicals into hydrogen peroxide, SOD plays a crucial protective role in cells. CAT in zebrafish similarly converts hydrogen peroxide into water and oxygen, preventing the toxic accumulation of this byproduct of cellular metabolism. Additionally, GPx reduces peroxides, including hydrogen peroxide and lipid peroxides, by utilizing glutathione, ensuring cells are shielded from oxidative stress. Furthermore, zebrafish have glutathione S-transferase (GST), an enzyme that helps detoxify harmful compounds by conjugating them with glutathione, enabling their excretion (Abbate et al. 2021; Cong et al. 2020). Additionally, exposure to zinc oxide nanoparticles (nano-ZnO) has been shown to induce excessive ROS production and oxidative DNA damage in zebrafish embryos, leading to apoptosis via mitochondrial pathways. Similarly, imidacloprid exposure results in increased ROS levels, lipid peroxidation, and DNA damage in zebrafish (Zhao et al. 2016). Furthermore, exposure to bis(2-ethylhexyl) phthalate (DEHP) has been reported to elevate oxidative stress markers and cause significant DNA fragmentation in zebrafish embryos, with damage confirmed through comet assay measurements (Boran et al. 2019).

These antioxidant enzymes in zebrafish function in ways analogous to those in mammals, working together to combat oxidative stress and maintain cellular health. The similarity in these systems makes zebrafish a useful model for studying oxidative stress and its relation to diseases such as cancer and aging in mammals.

Exposure to carcinogenic substances has been shown to induce the production of ROS in zebrafish, making them a valuable model for studying oxidative stress related to cancer. A study observed that arsenic exposure led to a marked reduction in the production of ROS in zebrafish larvae between days 4 and 11 post-fertilization (dpf). This reduction in ROS suggests that arsenic may interfere with normal cellular functions, particularly those related to oxidative stress, potentially affecting the development and immune system of the larvae. These results highlight the disruptive effects of arsenic on the larvae's ability to cope with oxidative stress, which may have broader implications for their overall development (Hermann and Kim 2005). In another study, researchers examined the protective effects of melatonin against arsenic-induced toxicity in zebrafish embryos. The study found that exposure to sodium arsenite (NaAsO₂) caused increased oxidative stress, leading to developmental cardiotoxicity and apoptosis in the zebrafish embryos. However, melatonin treatment significantly reduced ROS levels and apoptosis, thereby mitigating the harmful effects of arsenic exposure. These findings suggest that melatonin can effectively regulate oxidative stress and apoptosis, offering a potential therapeutic approach for counteracting arsenic-induced developmental toxicity in aquatic organisms (Yan et al. 2022).

Another research has demonstrated that exposure to erythrosine, a food dye considered a potential carcinogen, activates ROS generation in zebrafish embryos, leading to teratogenic effects and highlighting the role of oxidative stress in developmental abnormalities (Dharmar et al. 2019).

Another study focused on perfluorooctanesulfonamide, a compound from the per- and polyfluoroalkyl substances group, revealed that high concentrations induced oxidative stress markers and apoptosis-related gene expression in zebrafish. Additionally, glyphosate and glyphosate-based herbicides have been shown to affect zebrafish at various life stages, contributing to oxidative stress (David et al. 2024).

### Antioxidant Defense Mechanisms in the Body and the Effects of Oxidative Stress on DNA

Organisms have developed a complex, three-tiered antioxidant defense system to regulate ROS levels and protect against their harmful effects. The first and most powerful defense line consists of antioxidant enzymes such as SODs, CAT, and GPxs. This system works in concert to maintain ROS levels within physiological limits. ROS concentrations fluctuate over time—rising rapidly when needed for normal physiological functions and dropping sharply when oxidative damage threatens (Hagedorn et al. 2012; Jomova et al. 2024).

In zebrafish, somatic cells have electrophile-responsive genes that encode proteins to deactivate oxidants, preventing oxidative stress. When exposed to oxidative stress, zebrafish activate genetic pathways that produce antioxidants to mitigate the damage (Hagedorn et al. 2012).

Cancer development is a complex process that unfolds in three stages: initiation, promotion, and progression, all of which are influenced by oxidative stress and ROS. During initiation, normal cells acquire DNA mutations, creating cells with permanent genetic changes, often driven by ROS-induced DNA damage seen in cancer tissues. In the promotion stage, these mutated cells rapidly multiply due to increased cell growth or reduced apoptosis, with oxidative stress playing a key role by temporarily affecting genes that regulate cell division and death. ROS also impact crucial transcription factors like Nuclear factor kappa-light-chain-enhancer of activated B cells (NF-κB), Nuclear factor erythroid 2-related factor 2 (Nrf2), Hypoxia-inducible factor (HIF), and p53, which control cell growth and cancer development. Even low levels of oxidative stress can promote tumor growth by triggering cell division. In the progression stage, elevated ROS levels drive mutations, activate matrix metalloproteinases, damage surrounding tissues, and increase the genetic instability of cancer cells, boosting their metastatic potential. ROS also promote angiogenesis, aiding cancer spread (Ionescu et al. 2024).

## The Relationship Between Oxidative Stress and Genetic Factors in Cancer Development

### The Interaction Between Oxidative Stress and Genetic Mutations

As previously discussed, oxidative stress plays a crucial role in the development of genetic mutations that can lead to cancer. ROS, which are byproducts of cellular metabolism, can inflict damage on various cellular components, particularly DNA. This damage results in mutations in genes responsible for regulating essential cellular processes such as the cell cycle, apoptosis, and DNA repair mechanisms (Valko et al. 2006).

The types of DNA damage caused by oxidative stress include point mutations, deletions, and structural alterations. One common form of oxidative DNA damage is the formation of 8-OHdG, which can lead to GC to TA transversions, frequently found in tumor suppressor genes like Tumor protein 53 (TP53) (Cooke et al. 2003). The accumulation of these mutations is critical for the initiation of oncogenesis, as they compromise the integrity of the genome.

Point mutations, which involve a single nucleotide change in the DNA sequence, can significantly alter gene function. These mutations are especially critical when they occur in proto-oncogenes or tumor suppressor genes. A major way point mutations contribute to cancer is by activating proto-oncogenes. For example, a point mutation in the Ras gene can result in its persistent activation, leading to continuous signaling that drives cell proliferation even in the absence of external growth signals. This disruption of normal cell cycle regulation is a key feature of many cancers. This mechanism has been successfully recapitulated in zebrafish models, where liver-specific expression of mutant Kras^V12^ induces robust hepatocarcinogenesis, mirroring human pathology (Nguyen et al. 2011; Lin et al. 2025). Conversely, point mutations can also lead to the inactivation of tumor suppressor genes. A prominent example is the TP53 gene, which encodes the p53 protein, essential for DNA repair and cell cycle control. Mutations in TP53 impair its ability to repair DNA damage or trigger apoptosis in damaged cells, enabling the survival of mutated cells and allowing additional genetic alterations to accumulate, ultimately promoting cancer development (Vogelstein and Kinzler 2004). Zebrafish carrying TP53 mutations have been shown to spontaneously develop malignant peripheral nerve sheath tumors and other neoplasms, further supporting their relevance in modeling human cancer genetics (Berghmans et al. 2005b).

DNA deletions, which involve the removal of nucleotide sequences, can disrupt crucial genes and play a significant role in cancer development. When deletions occur in genes that control cell cycle progression or apoptosis, they can facilitate oncogenesis. For instance, the loss of one allele of the TP53 gene, often due to chromosomal deletions, results in haploinsufficiency, impairing the cell's ability to repair DNA damage. Without functional p53, cells with damaged DNA can continue to divide, accumulating more mutations that elevate the risk of cancer. Additionally, larger deletions can cause chromosomal instability, leading to further genetic alterations, including additional deletions, amplifications, or translocations. This increased genomic instability accelerates cancer progression by promoting the accumulation of mutations that drive tumor formation (Knudson 1971).

Structural alterations, including chromosomal translocations, inversions, and amplifications, can play a crucial role in oncogenesis by disrupting gene function or generating novel gene fusions. Chromosomal translocations are a prominent example of how structural changes contribute to cancer. These translocations can result in the formation of fusion genes, which often produce hybrid proteins with abnormal, cancer-promoting functions. A well-known example is the BCR-ABL fusion gene, which occurs due to a translocation between chromosomes 9 and 22, forming the Philadelphia chromosome. This fusion gene encodes a continuously active tyrosine kinase, driving uncontrolled cell division and leading to chronic myelogenous leukemia. In addition to translocations, structural alterations can also lead to the amplification of oncogenes, such as the myelocytomatosis oncogene (MYC). Amplification of MYC results in its overexpression, promoting excessive cell proliferation and significantly contributing to tumor formation (Hasty and Montagna 2014).

Furthermore, mutations in oncogenes and tumor suppressor genes are pivotal for cancer initiation and progression. For example, oxidative stress can activate oncogenes such as rat sarcoma virus (RAS), which is associated with unregulated cell proliferation. In the context of RAS-driven cancers, NRF2/antioxidant signaling plays a significant role in mediating chemoresistance and regulating altered metabolism. In addition, NRF2, a key transcription factor, has been shown to increase the resistance of cancer cells to chemotherapy (Nguyen et al. 2016). This is particularly evident in cancers with oncogenic mutations, such as Kirsten rat sarcoma viral oncogene homolog (KRAS), where NRF2 activation is linked to poor therapeutic outcomes (Mukhopadhyay et al. 2020). Studies indicate that oncogenic KRAS can regulate NRF2 expression, thereby promoting drug resistance by enhancing the antioxidant response and metabolic reprogramming that supports tumor growth (Zhao et al. 2020; Wang et al. 2008). Notably, Marques et al. (2024) investigated the activation of NRF2 during critical developmental windows in zebrafish embryos. Their study revealed tissue-specific changes in NRF2A protein levels and downstream effects on protein S-glutathionylation in the pancreatic islet and liver, highlighting the conserved role of NRF2 in zebrafish oxidative stress responses (Marques et al. 2024). Additionally, Marques et al. (2023) examined the developmental impacts of NRF2 activation by dimethyl fumarate (DMF) in zebrafish. They found that NRF2A activity influenced toxicity outcomes, with tissue-specific differences observed in NRF2A protein expression and protein S-glutathionylation in the pancreatic islet and liver during embryonic development (Marques et al. 2023).Furthermore, a study by Nakajima et al. (2011) documented the generation and characterization of a novel NRF2/ARE pathway biosensor fish, which exhibits dynamic spatio-temporal expression profiles during early developmental stages. This transgenic line responds to known NR2 pathway modulators, providing a valuable tool for studying NRF2 activity in zebrafish (Nakajima et al. 2011).

Concurrently, oxidative damage can inactivate tumor suppressor genes like TP53, disrupting their role in maintaining genomic stability and regulating apoptosis (Klaunig et al. 2010). Both RAS and TP53 are crucial genes involved in cancer development, and oxidative stress plays a significant role in their mutation and inactivation. RAS, a proto-oncogene, becomes an active oncogene when mutated, leading to uncontrolled cell proliferation. Oxidative stress can induce mutations in RAS, causing its continuous activation even in the absence of external growth signals, which promotes tumor formation. These mutations also alter cellular signaling pathways, including those that regulate metabolism and resistance to chemotherapy. For example, in cancers with mutations in KRAS, oxidative stress can activate NRF2 signaling, which enhances the survival of tumor cells by making them more resistant to treatment. On the other hand, TP53 is a tumor suppressor gene encoding the p53 protein, which is essential for maintaining genomic stability, regulating the cell cycle, and triggering apoptosis when DNA damage occurs. Oxidative stress can damage DNA, inactivating TP53 and impairing its ability to prevent the proliferation of cells with damaged DNA. As a result, these cells continue to divide, accumulating additional mutations that drive cancer progression. In many cancers, TP53 mutations allow genetically damaged cells to survive, further promoting tumor growth. The interplay between oxidative stress and these genes—both RAS and TP53—creates a conducive environment for oncogenesis, where mutations and disrupted signaling contribute significantly to cancer development (Thomas et al. 2022; Bansal 2023).

This interplay between oxidative stress and genetic mutations establishes a feedback loop that perpetuates cancer development. The feedback loop between oxidative stress and genetic mutations plays a crucial role in sustaining cancer development. ROS generated during oxidative stress can activate transcription factors like NRF2, which, in turn, induces the expression of antioxidant genes aimed at protecting the cell from damage. However, chronic oxidative stress can lead to persistent NRF2 activation, a hallmark of many cancers, which not only contributes to cellular survival but also enhances resistance to chemotherapy. This continuous activation creates a feedback loop where ROS perpetuate genetic mutations in critical genes like RAS and TP53. Mutations in RAS drive uncontrolled cell proliferation, while mutations in TP53 impair DNA repair mechanisms, allowing damaged cells to survive and divide. This interplay between oxidative stress and genetic mutations accelerates cancer progression by promoting further genetic instability, creating a cycle that is difficult to break and often results in aggressive tumor development (Pal and Firdous 2024).

**Fig. 2:**
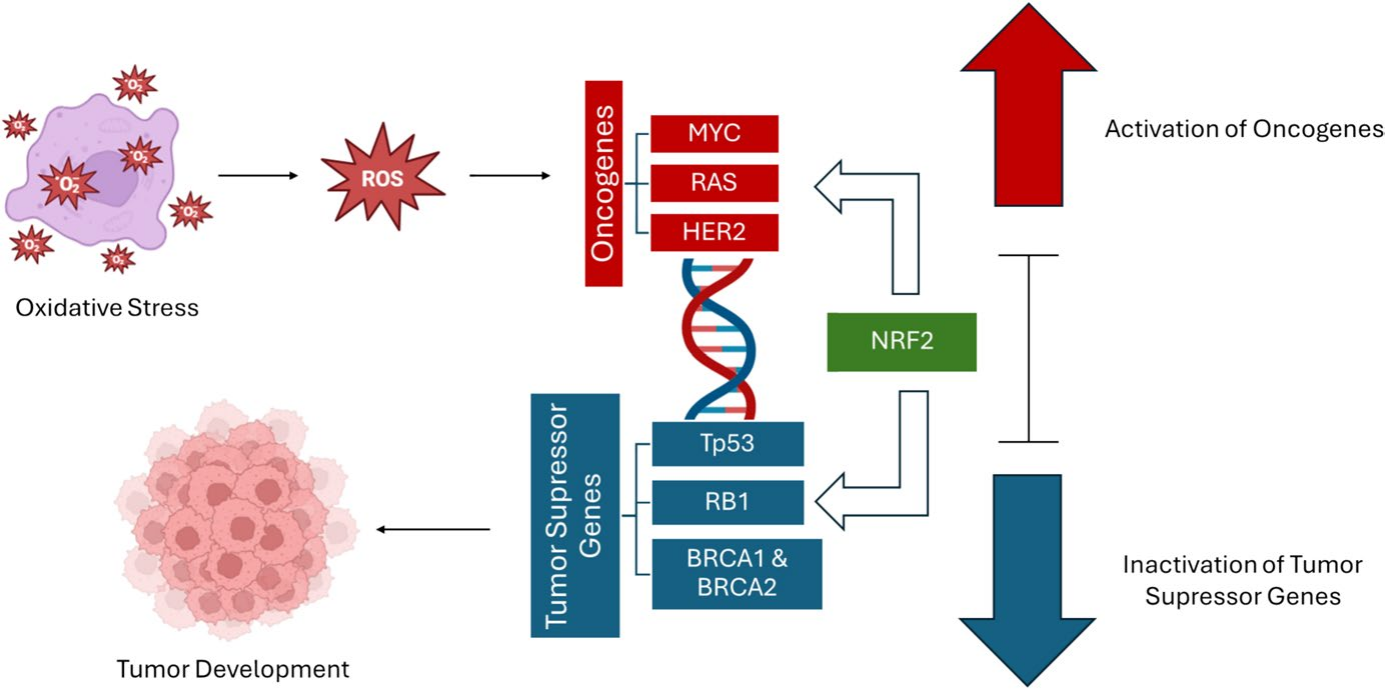
Diagram showing how oxidative stress via ROS activates oncogenes and inactivates tumor suppressor genes through NRF2, driving tumor formation and highlighting NRF2's dual role in cancer development. BRCA 1 – breast cancer 1; BRCA 2 – breast cancer 2; HER2—human epidermal growth factor receptor 2; MYC—myelocytomatosis viral oncogene; RAS—rat sarcoma virus; RB1—retinoblastoma 1; ROS – reactive oxygen species; TP53—tumor protein 53 (Created in BioRender. Ionescu, C. (2025) https://BioRender.com/a43k196)

An illustration has been included to demonstrate how oxidative stress, mediated by ROS, activates oncogenes through the transcription factor NRF2 while simultaneously inactivating tumor suppressor genes via the same pathway, ultimately contributing to tumor formation. This representation underscores the pivotal role of NRF2 in orchestrating the molecular mechanisms underlying cancer development (Fig. 2).

### The Influence of Oxidative Stress on the Expression of Oncogenes and Tumor Suppressors

Oxidative stress significantly influences the expression of oncogenes and tumor suppressor genes, which are vital for regulating cell growth and apoptosis. Elevated levels of ROS can activate signaling pathways associated with oncogenes, promoting tumor growth (Reuter et al. 2010).

The discovery and understanding of the genes responsible for cancer formation can contribute to developing specific therapies (Dang et al. 2016). Proto-oncogenes are normal genes that play a role in cell differentiation, growth, proliferation, and death (Hanahan and Weinberg 2011; Thomas et al. 2007; Zakiryanova et al. 2018; Gabay et al. 2014). In normal cells, proto-oncogenes play an essential role in regulating biological processes. They can function as growth factors, molecules involved in cellular signaling, and nuclear transcription factors (Kontomanolis et al. 2020). When proto-oncogenes undergo mutations or become hyperactive, they transform into oncogenes, which can trigger uncontrolled cell proliferation and, consequently, cancer development. Cancer is influenced by a few essential genes, a phenomenon known as "oncogene dependence". Although cancer cells contain numerous mutations and epigenetic changes, correcting a limited set of genes can significantly reduce tumor growth (Zakiryanova et al. 2018). Initially, oncogene dependence was viewed as a strictly cellular phenomenon, but recent research shows that immune mechanisms, including immune cells and cytokines, also contribute. Inactivation of oncogenes such as viral myelocytomatosis oncogene or Human Epidermal Growth Factor Receptor 2 (HER2) can trigger an anti-tumor immune response. Proto-oncogenes are also expressed in immune cells and may be involved in autoimmune diseases without requiring genetic mutations, suggesting activation mechanisms different from those in cancer (Zakiryanova et al. 2018).

Proto-oncogene MYC encodes transcription factors frequently activated in human neoplasms, and its overexpression can trigger tumorigenesis. Genetic alterations and epigenetic or post-translational mechanisms affecting MYC are common in cancers, varying depending on their type. These alterations include gene amplifications, chromosomal translocations, and mutations that increase MYC expression (Prior et al. 2012; Duffy et al. 2021). Dysregulation of the MYC gene is a common phenomenon in human cancer, being associated with more than half of malignant tumors (Table 1) (Vita and Henriksson 2006; Ahmadi et al. 2021).

**Table 1:** Key oncogenes and tumor suppressor genes associated with cancer development and progression

Genes type	Genes Associated with Cancer	Description	Type of cancer	Importance	References
Oncogenes	MYC	Universal transcription enhancer	Many types of cancer	It regulates almost every physiological process in a cell, including proliferation, cell cycle, metabolism, differentiation, and apoptosis	Gabay et al. 2014; Ahmadi et al. 2021; Shortt and Johnstone 2012)
	RAS	RAS proteins have a C-terminal CAAX sequence and are the most frequently mutated oncogenes in cancer	Pancreatic cancer Colorectal cancer Lung cancer	They are essential for transmitting signals from cell surface receptors to the nucleus, regulating cell growth, differentiation and survival	Hobbs et al. 2016; Kattan and Hancock 2020)
	HER2	Receptor located on the cell surface, which is part of the family of receptors for epidermal growth factors	Breast cancer Gastric cancer Colorectal cancer Lung cancer Ovarian cancer, etc	Essential role in the regulation of cell growth and division	Cheng 2024)
Tumor suppressor genes	TP53	Encodes the tumor suppressor protein p53	50% of human cancers	Its primary biological function involves protecting the integrity of the cell's DNA	Hernández Borrero and El-Deiry 2021)
	RB1	The first identified tumor suppressor gene whose mutational inactivation causes a human cancer and encodes the protein pRB	Pediatric retinoblastoma	Helps prevent tumor formation by strictly controlling cell division	Chinnam and Goodrich 2011)
	BRCA1	Genes essential for maintaining genome stability, having a crucial role in repairing damaged DNA through homologous recombination repair	Breast cancer Ovarian cancer	BRCA1/BRCA2 mutations prevent DNA repair, leading to the accumulation of genetic errors	Godet et al. 2017; Gorodetska et al. 2019)
	BRCA2				

*BRCA 1* breast cancer 1, *BRCA 2* breast cancer 2, *DNA* deoxyribonucleic acid, *HER2* human epidermal growth factor receptor 2, *MYC* myelocytomatosis viral oncogene, *RAS* rat sarcoma virus, *RB1* retinoblastoma 1, *p53* protein 53, *pRB* retinoblastoma protein, *TP53* tumor protein 53

Ras proteins, encoded by Harvey rat sarcoma viral oncogene homolog (HRAS), KRAS, and Neuroblastoma RAS viral oncogene homolog (NRAS), are frequently mutated proto-oncogenes in human cancers, playing a role in regulating cell proliferation and survival. They are activated by Guanine nucleotide exchange factor (GEF) and deactivated by GTPase-activating protein (GAP). Mutations at codons 12, 13, or 61 lead to constant activation of RAS, permanently binding guanosine triphosphate (GTP). Although RAS isoforms have similar sequences in key regions, their specific functions are determined by the hypervariable C-terminal region, which influences localization and cellular signaling (Dhanasekaran et al. 2022). Additionally, HER2, a receptor for epidermal growth factors, is associated with aggressive forms of cancer, particularly breast cancer (Cheng 2024).

These alterations create a favorable environment for cancer development, where oncogenes are overexpressed, and tumor suppressor functions are compromised, enhancing cellular proliferation and survival.

Oncogenes play a crucial role in regulating immune responses, including the activity of natural killer (NK) cells. Although it was assumed that immune changes increase as the disease progresses, they occur even in the early stages of tumors, with a significant reduction of NK cells in tumors. Thus, oncogenes promote both tumor development and the inhibition of immune responses against them (Zakiryanova et al. 2018).

Tumor suppressor genes, another class of genes, regulate essential functions such as cell growth and proliferation, DNA repair, and the induction of apoptosis. When these genes do not function correctly, there is an increased risk of uncontrolled cell growth, promoting the development of cancer. Loss-of-function mutations in tumor suppressor genes have been identified in numerous cancer types, including ovarian, lung, and colorectal cancers (Catherine and Appaji 2021). The most well-known gene is TP53, which encodes the tumor suppressor protein p53, known as the "guardian of the genome," and is essential for protecting DNA and controlling cellular processes such as cell cycle arrest, apoptosis, and DNA repair. p53 responds to cellular stresses by activating genes that control the fate of the cell. It is involved in over 50% of cancers, while the rest involve the inactivation of its pathway (Hernández Borrero and El-Deiry 2021). Furthermore, retinoblastoma 1 (RB1) is the first identified tumor suppressor gene and is crucial for controlling the cell cycle. It encodes the pRB protein, which inhibits cell cycle progression by binding to transcription factors that promote proliferation. Through this action, RB1 helps prevent tumor formation, and its mutational inactivation is particularly associated with pediatric retinoblastoma, a rare but aggressive cancer (Chinnam and Goodrich 2011).

On the other hand, BRCA 1 and BRCA2 are essential genes for DNA repair and maintaining genomic integrity. These genes encode proteins that play a critical role in homologous recombination repair, a mechanism that corrects errors in damaged DNA. Mutations in these genes lead to an inability to repair DNA, resulting in the accumulation of genetic errors that increase the risk of cancer, particularly breast and ovarian cancer. Studies have shown that individuals with mutations in BRCA1 or BRCA2 have a significantly higher risk of developing these types of cancer, highlighting the importance of these genes in cancer prevention (Godet et al. 2017; Gorodetska et al. 2019).

Mutations or overexpression of certain genes, such as MYC, can be triggered by environmental carcinogens like tobacco smoke or UV radiation. These factors disrupt cellular processes, including growth and metabolism, driving uncontrolled cell division and contributing to tumorigenesis. Similarly, mutations in RAS genes, particularly KRAS, are often the result of exposure to carcinogens like tobacco smoke (especially in lung cancer) or other environmental factors (such as diet and lifestyle in colorectal cancer). These mutations activate key signaling pathways, leading to unchecked cell proliferation (Cicenas et al. 2017).

In the case of HER2, overexpression can be caused by genetic amplifications or even viral oncogenes like HPV in cervical cancer. This overexpression stimulates cell growth signals excessively, helping cancer cells evade apoptosis and promoting tumor progression. TP53, often called the “guardian of the genome,” can also be mutated by carcinogens like UV radiation or tobacco smoke. When TP53 mutates, it fails to properly respond to DNA damage, allowing cells with damaged DNA to survive and proliferate, which increases cancer risk (Cicenas et al. 2017).

RB1 mutations, which prevent proper cell cycle regulation, can be induced by environmental carcinogens, especially in tissues like the retina and lungs. Without this control, cells divide uncontrollably, promoting tumor growth. BRCA1 and BRCA2 mutations also lead to defective DNA repair, which increases the risk of cancers such as breast and ovarian cancer. Exposure to carcinogens, particularly radiation, makes these defects worse, leading to genomic instability and a higher likelihood of tumor development (Godet et al. 2017; Cicenas et al. 2017).

### Genetic Mutations and Genomic Instability

Chromosomal rearrangements, tandem duplications, and deletions in cancer cells can generate fusion genes, which contribute to tumor evolution and progression. These events, common in the cancer genome, often precede observable malignant phenotypes (Glenfield and Innan 2021). Chromosomal deletions are characteristic of human cancers and have been associated with poor prognosis for over a century; however, their functions and mechanisms remained unclear until recently. Technical advances, such as cancer genomics, have opened new perspectives in the study of these deletions (Chen et al. 2018).

Many types of cancer are associated with chromosomal rearrangements, which can be simple or complex. Chromosomal rearrangements are structural modifications of chromosomes that can affect their organization, number, and function (Hasty and Montagna 2014). These rearrangements can play an important role in normal biological processes but are also involved in the development of various conditions, including cancer. Recent technological advancements have improved the detection of these rearrangements and have led to speculation about causal mechanisms, such as defective DNA repair and erroneous replication. A better understanding of these mechanisms may lead to the development of new therapies (Hasty and Montagna 2014).

Chromosomal instability involves the loss or gain of chromosomes or large DNA sequences, promoting the accumulation of oncogenic mutations. Aneuploidy, a form of chromosomal instability, refers to an unbalanced number of chromosomes resulting from mitotic errors during cell division, leading to the gain or loss of chromosomes (Chunduri and Barthel 2022). This can be lethal during embryonic stages in most metazoans, but in rare cases, human aneuploid embryos can survive, having severe pathological consequences, such as trisomy 21. Although aneuploidy also occurs in healthy cells, it is common in cancer cells and is a distinctive hallmark of cancer, often associated with chromosomal instability and improper segregation of chromosomes (Chunduri and Barthel 2022).

The loss of a chromosome leads to reduced mRNA levels and protein abundance for all genes located on the absent chromosome across all studied organisms. For example, when diploid cells lose a chromosome, the expression of genes should be reduced by half. Analyses of diploid and aneuploid blastocysts have shown decreased expression of genes on the monosomy. Additionally, a significant negative regulation of mRNA was observed for 64% of genes on chromosome 3p following its deletion. However, expression does not always align with gene copy number (Chunduri and Barthel 2022; Licciardi et al. 2018; Taylor et al. 2018).

Cells that loss or gain less than a complete set of chromosomes are aneuploid, and cells with a high rate of chromosome loss or gain exhibit chromosomal instability. Specific genotypes may be predisposed to instability, resulting in variable aneuploid descendants (Potapova and Gorbsky 2017). Cells can also be polyploid, having more than two centrioles, which can lead to the formation of multipolar spindles and improper segregation of chromosomes, generating aneuploids with a variable number of chromosomes. These segregation errors affect cellular physiology and the adaptability of organisms (Potapova and Gorbsky 2017).

Cytosine methylation is an important epigenetic process that involves the addition of a methyl group to the cytosine base in DNA. This process plays a crucial role in regulating gene expression, influencing genomic activity without altering the DNA sequence. In the context of cancer, cytosine methylation can have profound effects on the development and progression of the disease. There is a close link between DNA distribution and histone methylation, suggesting complex interactions between these epigenetic marks. DNA methyltransferase enzymes regulate DNA methylation. These processes engage in the etiology of congenital disorders and cancer, holding potential for the development of biomarkers and therapies (Li et al. 2021b).

### DNA Repair Mechanisms and Associated Defects

Preserving genetic information is essential for life, and mutagenesis contributes to evolution as well as the onset of cancer and other diseases. DNA is prone to chemical changes due to internal and external agents, and errors made by DNA polymerases during replication can cause mutations. However, cells have complex DNA repair mechanisms and cell cycle controls designed to minimize the negative effects of these damages (Chatterjee and Walker 2017). Cells respond to DNA damage by activating DNA damage response (DDR) pathways, which provide time to repair lesions. There are five main DNA repair pathways: base excision repair, nucleotide excision repair, mismatch repair, homologous recombination, and non-homologous end joining. When damaged DNA persists, apoptosis is activated to eliminate unstable cells. In many types of cancer, repair pathways and DDR are disrupted, increasing mutagenesis and genomic instability (Chatterjee and Walker 2017).

DNA damage plays a crucial role in cancer development, as it can generate abnormal nucleotides and mutations, contributing to genomic instability. DNA repair pathways correct damage caused by harmful agents, thus maintaining genomic stability. Inefficient DNA repair is essential for cancer evolution. A better understanding of these mechanisms can improve therapeutic interventions. DNA damage is divided into two categories: endogenous and exogenous. Endogenous damage arises from chemical reactions of DNA with water and ROS, while exogenous damage is caused by environmental agents such as UV and ionizing radiation, which can induce various types of DNA damage (Alhmoud et al. 2020).

Endogenous lesions occur from natural errors during DNA replication or from inappropriate interactions between enzymes that manipulate DNA. On the other hand, exogenous lesions are caused by external factors, such as radiation, which can generate highly reactive free radicals that cause breaks in the DNA strand. UV radiation is a major cause of skin cancer, affecting DNA both directly and indirectly. However, damaged DNA can be repaired. The enzyme O6-alkylguanine-DNA alkyltransferase helps repair lesions by transferring chemical groups, while α-ketoglutarate-dependent dioxygenases can correct other types of damage through oxidation. These repair mechanisms are essential for maintaining genome integrity and preventing cancer (Chatterjee and Walker 2017; Margison et al. 2007).

### Recent Studies on the Influence of Genetic Factors in Cancer Development in Zebrafish

In the past decade, cancer research has made significant progress, highlighting the genetic diversity of tumors. Mouse models have been predominantly used, but they have limitations in studying the early dissemination of tumors. However, zebrafish have become a valuable model due to their low cost, rapid external development, and the transparency of their embryos, which allows for the observation of tumor dynamics. This enables visualization of tumor interactions and genetic pathways like those in human cancer (Hason and Bartůnĕk 2019).

Recent studies have demonstrated the use of zebrafish for xenotransplantation of human cancer cells and small molecule screening, and genetic cancer models have been developed by introducing mutations in genes relevant to human cancers. These advances facilitate the study of tumor mechanisms and responses to treatments (Hason and Bartůnĕk 2019). As cancer research advances and becomes increasingly complex, innovative tools such as Clustered Regularly Interspaced Short Palindromic Repeats (CRISPR)/Cas9 offer new insights and solutions for oncological treatments, enabling not only the study of tumor mechanisms but also the development of personalized gene therapies. CRISPR/Cas9 is a revolutionary gene editing technology that allows for rapid and efficient correction of errors in the genome, as well as the activation/deactivation of genes (Redman and Ravussin 2011). In the context of hereditary cancer modeling, CRISPR/Cas9 has been effectively applied in zebrafish to create targeted mutations in key cancer-associated genes such as APC, TP53, and BRCA2 (Kobar et al. 2021). These models closely mimic human cancer phenotypes, such as intestinal tumor formation in APC-mutants and increased tumor susceptibility in TP53-deficient lines. Their transparency, rapid development, and genetic tractability make zebrafish an ideal system for real-time observation of tumor initiation and for preclinical screening of personalized therapies (Hason and Bartůnĕk 2019). Integrating CRISPR-generated zebrafish models into cancer research thus offers a powerful platform to bridge the gap between gene-level insights and clinical translation (Cornet et al. 2018). This technology has diverse applications in biomedical research, including the generation of cellular and animal models, functional genomic screens, and live imaging of the genome. Technology has demonstrated the ability to repair defective DNA in mice and modify human embryos. Potential clinical applications include gene therapy, treatments for infectious diseases, and the engineering of autologous materials for cancer treatment (Redman and Ravussin 2011).

## Zebrafish—A Model for Cancer Research

### Advantages of Using Zebrafish in Cancer Research

Zebrafish is a tropical species belonging to the Cyprinidae family and emerged as a model for scientific translational research in the late 1930s, used for its unique traits (Boix et al. 2020).

Firstly, an unique trait that this vertebrate possesses refers to the fact that humans and zebrafish exhibit a significant degree of genetic similarity, making zebrafish an excellent model for studying human biology and disease. Research indicates that approximately 71.4% of human genes have been identified to possess at least one orthologue in zebrafish, while around 82% of genes associated with human diseases also have corresponding zebrafish orthologues (Shen et al. 2023; Letrado et al. 2018; Astell and Sieger 2020).

However, while zebrafish provide substantial advantages, such as transparency of embryos and larvae, ease of drug administration, and rapid development, this model also has limitations in fully replicating the complexity of the human TME (Shimizu et al. 2023). For instance, zebrafish models are less effective in mimicking the stromal, vascular, and immune interactions present in human cancers, particularly in later tumor stages. To overcome these limitations, it is essential to integrate zebrafish-based systems with other advanced models, such as 3D cell cultures, organ-on-chip technologies, and tissue explant cultures (Crouigneau et al. 2024). These systems enhance the physiological relevance of preclinical cancer models by enabling better replication of the TME and cell–cell interactions.

3D cell cultures allow for the development of spheroids or organoids that more accurately represent tumor architecture and hypoxic gradients, which are often missing in traditional 2D cultures (Urzì et al. 2023). Likewise, organ-on-chip technologies mimic the dynamic interactions between tissues, extracellular matrices, and fluids, providing a platform to study cancer processes such as metastasis, immune infiltration, and drug responses in a microfluidic environment (Imparato et al. 2022). Tissue explant cultures, derived directly from patient samples, retain the structural and cellular complexity of tumors, making them invaluable for translational research and personalized medicine approaches (Piwocka et al. 2024).

To further increase model complexity, recent studies have demonstrated the feasibility of direct transplantation of patient-derived tissue fragments into zebrafish embryos, a method referred to as ZF “Avatars”. These models can reflect patient outcomes and support co-clinical trials, marking a step toward precision medicine. However, their application to preclinical studies remains challenging due to the limited availability of tissue and complex sample characterization. In this context, the transplantation of more controllable 3D models within zebrafish embryos could offer a balance between the advantages of a simple in vivo system and the preservation of the “spatio-temporal features” of tissue-mimetic cultures. This gradual integration advances the goal of creating a reliable and complex in vitro/in vivo cancer organ model (Miserocchi et al. 2023).

By combining zebrafish with these advanced systems, researchers can bridge the gap between in vitro and in vivo studies, enhancing the predictive value of preclinical cancer models. This integration not only helps to address the limitations of zebrafish in recreating a realistic TME but also increases the overall complexity and reliability of cancer research tools.

Another key advantage of zebrafish is that their embryos and larvae are transparent. This unique characteristic allows scientists to directly observe embryogenesis in real time and utilize optical imaging techniques for in vivo cellular and subcellular studies (Shen et al. 2023). In the context of cancer research their transparency is crucial for visualizing the earliest stages of host-tumor interactions. This feature allows to conduct real-time studies of important processes, such as angiogenesis and metastasis, as well as to explore how tumors interact with the innate immune system (Martinez-Lopez et al. 2021).

This remarkable model organism was also used for various biological studies, particularly due to their unique life cycle and reproductive characteristics. On average, zebrafish live for about 3 years and can reach up to 5 years under optimal laboratory conditions (Kishi et al. 2009). They are notable for their reproductive capabilities, characterized by asynchronous ovaries that contain follicles at different developmental stages alongside mature eggs The maturation and growth of oocytes take approximately 10 days, and under laboratory conditions, female zebrafish can spawn year-round (Gioacchini et al. 2010). Amazingly, young females achieve sexual maturity in about 2 months and can produce several hundred eggs every two weeks, with a quick ovulation recovery period of just 1 to 2 weeks. The fertilization of eggs occurs externally in water, allowing the transparent embryos to develop outside the mother. This external development enables researchers to conduct detailed observations, making zebrafish an ideal model for studies in developmental biology (Liu et al. 2021). They exhibit remarkable fertility, far surpassing that of mice. After 48 h of fertilization, zebrafish embryos develop fully functional organ systems, including the heart, intestines, and blood vessels. Additionally, zebrafish possess all the key organs necessary for metabolism, making them a valuable model for studying various human metabolic disorders. Successful mating can yield hundreds of fertilized eggs, which hatch approximately 72 h after fertilization. These eggs develop rapidly, transitioning from embryos into larval fish. By 90 days post-fertilization, zebrafish are fully matured and ready to reproduce (Issac et al. 2021; Singhal et al. 2023). The costs associated with zebrafish husbandry are significantly lower compared to those for mammals, primarily due to their minimal maintenance requirements. This economic advantage makes them an attractive option for research facilities. Furthermore, zebrafish have the ability to absorb substances directly from their aquatic environment, which facilitates the administration of drugs dissolved in water. This allows researchers to easily introduce and study various compounds in a controlled setting, enhancing the versatility of zebrafish as a model organism in biomedical research (Letrado et al. 2018).

### Applications in Cancer Research on Zebrafish

**Fig. 3:**
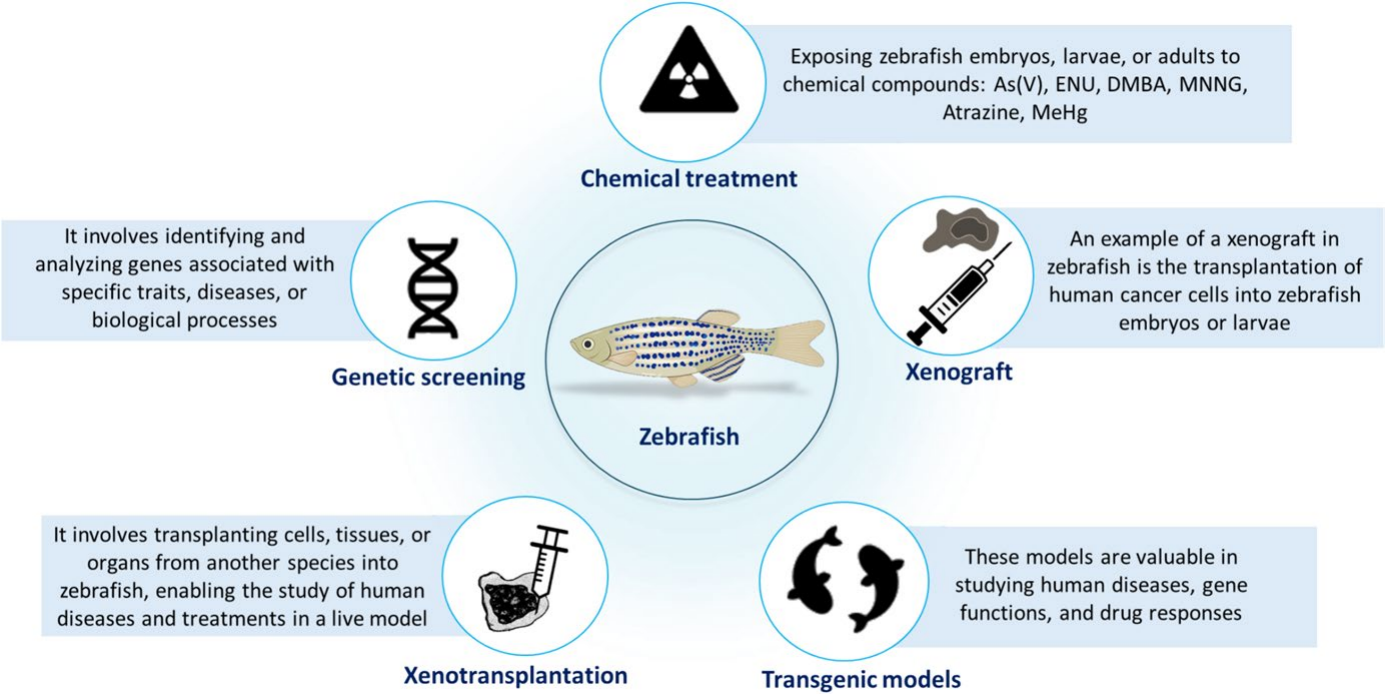
The Main Application of Zebrafish in Cancer Research. As(V)-sodium arsenate; ENU-N-ethyl-N-nitrosourea; DMBA-7,12-Dimethylbenz[a]anthracene; MNNG- N-Methyl-N’-nitro-N-nitrosoguanidine; MeHg- Methylmercury (Created in BioRender. Ionescu, C. (2025) https://BioRender.com/w37r782)

In Fig. 3 we presented the main applications in cancer research involving zebrafish. Researchers initially observed the spontaneous development of diseases, including cancer, in adult zebrafish maintained under laboratory conditions. Subsequent studies revealed that exposure to specific mutagens could induce a variety of tumors in zebrafish, closely resembling those found in humans in terms of both morphology and signaling pathways. The most frequent sites for spontaneous tumor formation include the gut, thyroid, and liver, with less frequent occurrences in the brain, blood vessels, and gills. Building on the findings of spontaneous tumor development in zebrafish, chemical methods have been developed to systematically induce cancer. This process involves exposing zebrafish to carcinogens dissolved in water for varying durations, taking advantage of their high tolerance to chemical exposure. Specific mutagens, such as 7,12-dimethylbenz(a)anthracene, have been shown to induce a wide range of tumors, from epithelial tumors in the intestines to mesenchymal tumors in blood vessels. Additionally, N-nitrosodiethylamine is known to induce pancreatic and liver carcinomas, while N-nitrosodimethylamine specifically targets the liver, resulting in tumor formation (Huiting et al. 2015). Other various carcinogenic compounds are capable of inducing cancer in multiple organs, including DMBA, diethylnitrosamine (DEN), ENU, and MNNG (Zhao et al. 2015).

The first instance of xenotransplantation of human cells into zebrafish was reported in 2005 by Lee et al., who successfully introduced human metastatic melanoma cells into zebrafish embryos at the blastula stage. Since then, numerous studies have shown that zebrafish embryos can support the engraftment of various human tumor cells, marked by fluorescent labels, for the study of processes such as tumor-induced angiogenesis, metastasis, cancer cell invasion, and drug screening. Zebrafish embryos are especially advantageous due to their transparency, ease of drug administration, and permeability to small molecules. They also allow for transient gene expression modulation through morpholino or mRNA injection. The ideal stage for xenotransplanting human cancer cells is around 2 days post-fertilization (dpf), as many embryos can be injected within a short period, ensuring a large sample size for analysis. Researchers have explored different injection sites like the yolk sac, bloodstream, or perivitelline space, and a range of cancers, including melanoma, leukemia, brain, breast, and pancreatic cancers. However, the utility of zebrafish embryos is limited to a week post-xenotransplantation due to their rapid development. Nonetheless, they remain a valuable platform for studying cancer biology and testing therapeutic interventions (Zhao et al. 2015).

Xenograft transplantation involves transferring living cells between different species. Zebrafish serve as a valuable model for studying human cancer cell transplantation. In this model, transplanted human cancer cells not only survive but also migrate and interact with the host environment. Zebrafish xenograft studies have been successfully used to investigate a wide range of human cancers, including melanoma, breast cancer, and leukemia, among others. The zebrafish embryo-larva stage provides several advantages over other xenograft models (Gamble et al. 2021).

One particularly exciting application is the use of zebrafish for patient-derived xenografts (zPDX). The zPDX model offers a highly efficient platform for studying cancer biology and therapy. By transplanting tumor cells or small fragments of human tumors into zebrafish embryos, researchers can replicate key features of the human TME, including cellular diversity and interactions with stromal components. Using tissue fragments rather than dissociated cells preserves the structural integrity of the tumor, enhancing the model's physiological relevance (Franco et al. 2022; Shen et al. 2020).

Transplanting entire tumor samples from patients into zebrafish has proven effective in replicating the metastatic behavior of the original tumors. For example, when pancreatic tumor fragments were implanted into the yolk sac of zebrafish, the tumors exhibited similar invasive and metastatic behaviors as those observed in the patient. This method is particularly advantageous because it requires only small quantities of tumor tissue, enabling the execution of numerous experiments using just a single biopsy sample (Gamble et al. 2021; Marques et al. 2009).

A key factor in these models is the choice of injection site, as it determines the type of cancer dynamics that can be studied. For example, the perivitelline space (PVS), located between the chorion and the yolk, is one of the most commonly used sites for xenograft injections, particularly in early zebrafish embryos. This location is ideal for monitoring tumor growth and proliferation because it offers a nutrient-rich environment, and the transparency of zebrafish allows for real-time observation of tumor development (Zhong et al. 2023).

Another frequently used injection site is the yolk sac, which provides generous space for tumor cells to grow. It is the most commonly used injection site in zebrafish xenograft models, mainly because of its large size, ease of access, and capacity to hold a substantial number of tumor cells. Additionally, it provides a nutrient-rich environment that supports cell proliferation, making it an ideal location for studying tumor growth and therapeutic responses (Shen et al. 2023). It is particularly beneficial for investigating early stage cancer development and how tumors interact with the vasculature, especially in the context of angiogenesis.

For studying metastasis, researchers often inject tumor cells into the caudal vein, which mimics how cancer cells enter the bloodstream and travel to distant organs. This method is invaluable for studying the spread of cancer and for testing anti-metastatic treatments. The ability to track the movement of tumor cells throughout the zebrafish’s body enables a more detailed understanding of cancer dissemination (Röss et al. 2023).

Finally, orthotopic injection is used when researchers aim to mimic the tumor's native environment. By directly injecting tumor cells into tissues such as the muscle or brain, we can study how cancers behave in the same organ where they would naturally develop (Lunina et al. 2024; Larsson et al. 2022).

Each of these injection sites provides unique advantages, allowing zebrafish xenografts to be a versatile model for studying various aspects of cancer biology and for testing potential cancer therapies. Zebrafish have also become an important model for cancer-related genetic screening. Scientists employ both forward and reverse genetic methods to uncover genes involved in cancer. In forward genetics, random mutations are introduced, and cancer-causing mutations are identified through observation. Reverse genetics, on the other hand, specifically modifies known genes, such as oncogenes or tumor suppressors, to study their role in cancer development. These approaches help researchers understand the genetic mechanisms behind cancer and assist in identifying potential therapeutic strategies (Raby et al. 2020).

In cancer research, zebrafish transgenic models consist of modified genetic lines where specific cancer-associated genes, including oncogenes and tumor suppressors, are altered. These models provide valuable insights into real-time inflammatory responses, particularly how immune cells such as macrophages and neutrophils are attracted to pre-neoplastic cells, which have the potential to transform into cancer. These immune cells, similar to those found in mammalian systems, are vital in the early stages of tumor development as they can facilitate tumor progression. Research involving zebrafish has deepened the understanding of the adaptability and roles of these immune cells during the onset of cancer. Moreover, advancements in genetic manipulation and imaging technologies for zebrafish have significantly improved cancer research, enabling more precise observations of inflammation's role in tumor formation and aiding in the development of targeted therapies (Elliot et al. 2020).

### Types of Cancer Induced by Exposure to Chemicals in Zebrafish

Cancer is characterized by the uncontrolled proliferation of abnormal cells and is classified according to the tissue or organ in which it occurs. There are over 200 types of cancer, organized based on their site of origin and similarities to certain histological cells within the respective tissue. Recent technological advances have facilitated the detailed analysis of the molecular composition of various forms of cancer (Brown 2021; Krieghoff-Henning et al. 2017). Cancer exhibits characteristic features such as aberrant signal transduction, which leads to uncontrolled cell proliferation, metastasis, inhibition of apoptosis, and angiogenesis (Kontomanolis et al. 2020).

Various chemical compounds have been studied for their potential to induce cancer in zebrafish, providing insights into tumorigenesis relevant to human health. Below are some notable examples of these compounds, the types of cancer they induce, and the corresponding sources (Table 2).

**Table 2:** Carcinogenic Effects of Various Chemicals and Their Predisposition to Induce Tumors in Zebrafish

Chemicals	Location	Cancer type	Predisposition	Mechanistic Role	References
As(V)	Bladder, liver, kidney, lung	Bladder cancer, liver cancer, kidney cancer, lung cancer	Predisposition to tumors	It induces oxidative stress and DNA damage, leading to mutations and alterations in cell signaling pathways	Xu et al. 2013)
ENU	Liver, neuroblastoma, sarcomas	Liver cancer, neuroblastoma, sarcomas	Predisposition to tumors	It is an alkylating agent that causes DNA mutations, disrupting gene function and promoting tumorigenesis	Solnica-Krezel et al. 1994)
DMBA	Skin, liver, kidney	Skin cancer, liver cancer, kidney cancer	Predisposition to tumors	It is a potent carcinogen that promotes DNA adduct formation and mutations, initiating tumor development	Spitsbergen et al. 2000)
MNNG	Squamous cell carcinomas, papillomas, gastrointestinal tract	Squamous cell carcinomas, papillomas	Predisposition to tumors	It induces mutations by methylating DNA, leading to changes in gene expression and cancer development	Shen et al. 2023)
Atrazine	Neuroendocrine system, reproductive organs	Neuroendocrine cancer, reproductive cancer	Predisposition to tumors	It disrupts endocrine signaling, leading to hormonal imbalances that promote tumor formation	Wirbisky et al. 2016)
MeHg	Neural tissues	Neural tissue cancer	Predisposition to tumors	It causes oxidative stress and disrupts cellular function in neural tissues, fostering cancer	Carvan et al. 2017)

As(V)-sodium arsenate; ENU-N-ethyl-N-nitrosourea; DMBA-7,12-Dimethylbenz[a]anthracene; MNNG- N-Methyl-N’-nitro-N-nitrosoguanidine; MeHg- Methylmercury

As(V) has been linked to the development of tumors in the bladder, liver, kidney, and lung of zebrafish. The study by Xu et al. (2013) highlights the molecular pathways and biomarkers associated with arsenic exposure, particularly focusing on liver effects in zebrafish (Xu et al. 2013). A study by Hallauer et al. (2016) investigated the effects of chronic arsenic exposure in zebrafish over six months at concentrations ranging from 50 to 300 ppb. The study found no visible tumor formation under these exposure conditions. However, the zebrafish exhibited neurological dysfunction, evidenced by reduced locomotive activity, and increased oxidative stress, indicated by elevated levels of the SOD protein in the eye and liver. Furthermore, progeny of arsenic-treated parents displayed a significant reduction in body weight compared to controls (Hallauer et al. 2016).

Exposure to ENU has been associated with tumorigenesis in the liver, neuroblastoma, and various sarcomas. Research conducted by Solnica-Krezel et al. (1994) details the efficiency of recovering ENU-induced mutations in zebrafish, showcasing its utility in cancer genetics (Solnica-Krezel et al. 1994; Raby et al. 2020). A pivotal study by Beckwith et al. (2000) demonstrated that adult male zebrafish treated with ENU developed skin papillomas at a 100% incidence rate within one year. The lesions ranged from epidermal hyperplasia to flat and pedunculated papillomas, with some exhibiting angiogenesis. Additionally, two cavernous hemangiomas and a malignant peripheral nerve sheath tumor were observed in the treated fish, while none of the untreated controls developed tumors (Beckwith et al. 2000).

These studies highlight the differential tumorigenic responses in zebrafish to ENU and arsenic exposures. While ENU has been shown to induce neoplasia effectively, arsenic exposure under the studied conditions did not result in tumor formation but did cause other physiological and generational effects.

DMBA is known to induce tumors in the skin, liver, and kidneys of zebrafish. Spitsbergen et al. (2000) conducted a study examining neoplasia in zebrafish treated with DMBA at different developmental stages, providing valuable data on its carcinogenic effects (Spitsbergen et al. 2000).

MNNG exposure has been shown to result in squamous cell carcinomas, papillomas, and gastrointestinal tract tumors in zebrafish. Research by Shen et al. (2023) discusses the application of zebrafish as a model for evaluating anti-cancer activities and toxicity testing related to natural products (Shen et al. 2023).

Atrazine is linked to alterations in the neuroendocrine system and reproductive organs. The work by Wirbisky et al. (2016) reveals that embryonic exposure to atrazine results in changes to genes associated with neuroendocrine function in adult male zebrafish (Wirbisky et al. 2016).

Exposure to methylmercury has been correlated with abnormalities in neural tissues. A study by Carvan et al. (2017) found that mercury-induced epigenetic changes can lead to transgenerational neurobehavioral abnormalities in zebrafish (Carvan et al. 2017).

Although not all chemicals have been conclusively proven to be carcinogenic, many have been tested and shown to possess a high potential for inducing cancer. Research indicates that various compounds, including sodium arsenate, N-ethyl-N-nitrosourea, and others, demonstrate significant carcinogenic properties in zebrafish models, making them valuable for studying tumorigenesis and cancer mechanisms. These studies provide insights into how these chemicals interact with biological systems and contribute to cancer development, even if definitive proof of their carcinogenicity is still under investigation.

For instance, while ENU is widely recognized for its mutagenic effects, not all mutations lead directly to cancer, but the compound's ability to induce a variety of tumors highlights its high potential for carcinogenic activity (Solnica-Krezel et al. 1994; Raby et al. 2020). Similarly, while sodium arsenate is linked to tumors in several organs, further research is needed to fully understand its mechanisms of action and long-term effects on zebrafish and, by extension, human health.

Overall, these compounds continue to be tested in zebrafish models for their cancer-inducing capabilities, providing a critical understanding of environmental carcinogens and their impact on health.

These findings underscore the importance of zebrafish as a model organism for understanding the effects of environmental chemicals on cancer development. The cited research not only aids in identifying cancer-related pathways but also contributes to broader implications for human health.

## Behavioral Tests Conducted on Zebrafish in Cancer Studies

### The Importance of Behavioral Tests in Evaluating Health Status and Nervous System Function

Behavioral tests using zebrafish have emerged as a vital tool in assessing health status and the functioning of the nervous system. These tests provide insights into various aspects of behavior, including anxiety, social interactions, and cognitive abilities, making zebrafish an invaluable model for neuroscience research.

Zebrafish are increasingly utilized in behavioral neuroscience studies. The behavioral repertoire of zebrafish includes complex social behaviors, learning, and memory, which are crucial for understanding neuropsychiatric disorders (Ogi et al. 2021).

The Novel Tank Diving Test assesses anxiety-like behavior by measuring how long zebrafish spend at the bottom versus the top of a tank when placed in a new environment. A longer latency to explore the upper regions indicates higher anxiety levels (Kysil et al. 2017).

Similar to rodent models, this assay evaluates preference based on exposure to rewarding or aversive stimuli. Zebrafish demonstrate a preference for areas associated with positive stimuli (e.g., food or drugs) over those linked to negative experiences (Abril-De-Abreu et al. 2015).

Shoaling Behavior Tests examine social interactions by observing how zebrafish respond to conspecifics. Factors such as group size and environmental novelty can influence shoaling behavior, providing insights into social anxiety and stress responses (Engeszer et al. 2007). Mirror Biting Test measures aggressive behavior by introducing a mirror to solitary zebrafish. The fish’s response can indicate social dominance and aggression levels (Pham et al. 2012).

Thus, by quantifying anxiety-like behaviors through various tests, researchers can assess the impact of environmental stressors or pharmacological agents on nervous system function. For example, anxiolytic drugs can be tested for their effectiveness in reducing anxiety-like behaviors in zebrafish. Also, tests such as conditioned place preference and novel object recognition help assess learning and memory abilities, which are critical to understanding cognitive impairments related to neurological disorders.

On the other hand, understanding social dynamics through shoaling and aggression tests offers insights into neurodevelopmental disorders that affect social interactions in humans, such as autism spectrum disorders (Ogi et al. 2021).

Thus, behavioral testing in zebrafish is a powerful approach for evaluating health status and nervous system function. These tests provide critical insights into anxiety, cognition, and social behavior, facilitating the study of neuropsychiatric disorders. As research continues to evolve, zebrafish will play an increasingly important role in understanding complex behaviors and developing therapeutic interventions for human health issues.

Zebrafish have become a prominent model organism for the study of behavior and social interactions, particularly in the context of neuropsychiatric disorders. Sociability tests, which assess how zebrafish interact with conspecifics, provide insights into the effects of oxidative and genetic stress on social dynamics (Schumann et al. 2023). This understanding is crucial for elucidating the mechanisms underlying social behavior and its implications for health.

Sociability tests usually involve placing a focal zebrafish in a controlled environment where it can interact with or observe other zebrafish (Kim et al. 2018). Tests often use setups similar to those used in rodent studies, such as three-chamber arenas (Barba-Escobedo and Gould 2012). In these tests, time spent near social stimuli—such as groups of conspecifics—versus nonsocial stimuli is measured to determine social preference (Barba-Escobedo and Gould 2012).

In zebrafish, oxidative stress can significantly affect behavior and social interactions. Studies have shown that exposure to oxidative stressors can alter the sociability of zebrafish, often leading to increased anxiety-like behaviors (Cook et al. 2023). For example, zebrafish under oxidative stress may spend less time interacting with conspecifics and more time in isolation, indicating impaired social functioning (Schumann et al. 2023).

Research indicates that oxidative stress can disrupt neurotransmitter systems involved in social behavior, such as the serotonin and dopamine pathways. Consequently, sociability tests may be essential in assessing how oxidative stress influences social interactions and general health (Apirajkamol et al. 2020).

Genetic stress refers to the impact of genetic mutations or alterations on the physiology and behavior of an organism (Buchanan and Lovallo 2019). In zebrafish, genetic changes—either through targeted gene editing or naturally occurring mutations—can provide insights into the genetic basis of social behavior (Ribeiro et al. 2020). Sociability tests can reveal how specific genetic changes affect social interactions.

For example, studies have shown that genetically modified zebrafish exhibit altered shoaling behaviors that are essential for survival in natural environments (Buske and Gerlai 2011). These changes can lead to variations in aggression, mating preferences, and overall sociability (Lee and Beery 2022; Bath et al. 2020). By comparing the sociability of wild-type and genetically engineered zebrafish, researchers can identify genes associated with social behavior and their potential implications for neurodevelopmental disorders (Gemmer et al. 2022; Dreosti et al. 2015).

The interplay between oxidative and genetic stress is particularly relevant when assessing social interactions in zebrafish (Gemmer et al. 2022). Thus, assessing how oxidative stress alters sociability in genetically modified zebrafish may reveal critical interactions between environmental factors and genetic predispositions (Clark et al. 2011; Aluru 2017).

In addition, identifying specific behavioral changes associated with oxidative or genetic stress may lead to targeted therapies for managing social deficits in associated human disorders (Hong and Iakoucheva 2023). Conclusively, zebrafish sociability assays serve as a powerful tool for assessing social interactions in the context of oxidative and genetic stress. By examining how these stresses influence social behavior, researchers can gain valuable insight into the mechanisms underlying neuropsychiatric disorders. As zebrafish continue to be used in behavioral neuroscience research, their role in understanding the complexity of social interactions will undoubtedly expand, providing possible avenues for therapeutic interventions.

Lately, the interaction between spatial memory and neuronal damage has attracted significant attention, especially in the context of carcinogenesis. Thus, understanding how cancer-related processes influence cognitive functions, particularly spatial memory, may provide insights into the broader implications of neural health in cancer patients.

Spatial memory is a critical cognitive function that allows organisms to navigate their environment. It is mediated primarily by the hippocampus, a region of the brain sensitive to various forms of neuronal damage (Shetty 2014). Research indicates that conditions such as chronic inflammation and oxidative stress, often seen in cancer patients, can lead to significant impairments in spatial memory.

Oxidative stress and inflammation are among the main mechanisms of neuronal damage. Thus, increased oxidative stress has been linked to neuronal damage and cognitive decline in various neurodegenerative diseases, including those associated with cancer. For example, studies have shown that oxidative stress can induce DNA damage, leading to cognitive impairments such as spatial memory deficits (Foret et al. 2024). Meanwhile, chronic inflammation has been associated with microglia activation, which can exacerbate neuronal damage. In cancer models, such as those involving Tusc2 knockout mice, inflammation correlates with significant spatial memory deficits due to altered immune responses in the brain.

The relationship between carcinogenesis and cognitive function is complex. Neuronal damage induced by tumor progression or treatment (e.g., chemotherapy) can lead to cognitive impairment. For example, vincristine-induced lesions have been shown to cause severe cognitive deficits in spatial memory tasks (Meléndez et al. 2020).

In addition, human neural stem cell (hNSC) transplantation studies suggest possible therapeutic avenues for restoring spatial memory by modulating inflammatory pathways and improving neuronal health (Chen et al. 2023).

Relatedly, research using transgenic mouse models has demonstrated that interventions targeting inflammation or oxidative stress can restore spatial memory capabilities even when significant neuronal damage is present. For example, hNSC transplantation not only improved spatial memory but also normalized dysregulated gene expression associated with inflammation (Chen et al. 2023).

In addition, tests such as the Morris water maze are essential in quantifying spatial memory deficits in animal models subjected to carcinogenic processes. These assessments reveal a direct correlation between the extent of neuronal damage (e.g., amyloid plaque burden) and performance on spatial memory tasks (Dabrowska and Wiczkowski 2017).

The correlation between spatial memory and neuronal damage in the processes of carcinogenesis highlights an essential area of research bridging oncology and neuroscience. Understanding these relationships may pave the way for new therapeutic strategies aimed at attenuating cognitive decline in cancer patients through targeted interventions that address both inflammatory and oxidative stress pathways. Further exploration of these mechanisms will be crucial for the development of effective treatments that protect cognitive functions during cancer treatment and progression.

### The Relevance of Behavioral Tests in Studying the Effects of Carcinogenic and Non-carcinogenic Substances on Cognitive Functions in Zebrafish

Zebrafish have emerged as a pivotal model organism for studying the effects of cancer on cognitive functions due to their unique biological and behavioral characteristics. This text explores the significance of behavioral tests in understanding how cancer influences cognitive processes in zebrafish, highlighting their utility in both basic research and therapeutic development.

Zebrafish possess a range of neurobehavioral traits that are comparable to those of higher vertebrates, including humans. Their cognitive abilities, such as learning, memory retention, and decision-making, can be assessed through various behavioral tests. Recent studies have indicated that cancer can significantly affect cognitive functions in zebrafish. For instance, research has shown that zebrafish exhibiting pessimistic cognitive bias—often linked to stress—are more susceptible to tumor development. This suggests a correlation between cognitive judgment biases and disease resilience, where optimistic zebrafish demonstrate lower tumorigenesis rates (Devidas et al. 2021). Such findings underscore the potential of using behavioral assessments to evaluate the cognitive impact of cancer therapies.

Behavioral tests serve multiple purposes in cancer research, for example zebrafish models are employed to screen compounds that may protect against cognitive decline induced by cancer treatments. The rapid development and genetic manipulability of zebrafish facilitate high-throughput screening processes (Devidas et al. 2021; Clevenger et al. 2024).

#### The Light–Dark Test (LDT)

Protocol typically involves placing zebrafish into an arena that has two distinct areas: one dark and one light. The time spent in each area is recorded to assess anxiety-like behavior. An increase in the time spent in the dark zone is generally interpreted as heightened anxiety or stress, as zebrafish typically prefer the light. The test is used to measure how exposure to substances, like chemicals or drugs, alters their behavior in response to environmental stressors. The results provide insights into the anxiety levels or stress responses of the fish. It has been used across multiple studies to assess anxiety-like behavior, with substances like BaA, atrazine, and CuSO4 showing altered light–dark preferences. This test predominantly uses adult zebrafish, and the primary effect observed is increased anxiety, as reflected by prolonged time spent in dark areas (Zhang et al. 2024; Steinberg et al. 1995; Maximino et al. 2011; Sackerman et al. 2010; Haverroth et al. 2015).

#### Social Behavior Test (SBT)

In this test, zebrafish are placed in a social environment with conspecifics, and their interactions are monitored. The protocol typically involves measuring the amount of time spent in proximity to other fish, the frequency of interactions, and how zebrafish respond to social stimuli. A decrease in social interactions or altered group dynamics can indicate disruptions in social behavior, often due to chemical exposure. For example, BaA exposure at early developmental stages caused disruptions in social behavior across larvae, juvenile, and adult zebrafish. Similarly, arsenic exposure also resulted in reduced social interactions, observed in both larvae and adults. These findings underscore the persistent effects of early toxicant exposure on zebrafish behavior (Hallauer et al. 2016; Zhang et al. 2024; Wang et al. 2023).

#### Shoaling Test (ST)

The ST assesses zebrafish social behavior by placing them with a group of conspecifics. The fish's tendency to swim together (shoal) is observed, and changes in the cohesiveness of the group, such as looser or disrupted shoals, are recorded. This test can reveal changes in social coordination and group behavior caused by environmental factors or exposure to toxic substances. BaA exposure led to disrupted shoaling, indicative of social dysfunction. This test, done on larvae, revealed looser groups and altered group dynamics, suggesting that exposure to toxicants during embryonic stages has long-lasting consequences on neural pathways responsible for social behavior coordination (Zhang et al. 2024).

#### Open Field Test (OFT)

In the OFT, zebrafish are placed in a novel arena with an open, unstructured space. The test measures locomotor activity, with particular attention to thigmotaxis (the tendency to stay near the edges of the arena). Increased time spent near the edges suggests anxiety-like behavior, while exploration of the central area indicates normal behavior. This test helps assess general activity levels and anxiety responses. Several substances, including methylmercury, nicotine, and arsenic, induced increased thigmotaxis, where zebrafish exhibited anxiety-like behavior by spending more time near the tank’s edges. This test is usually performed on larvae and adults, with a consistent trend toward reduced exploratory behavior in exposed zebrafish (Hallauer et al. 2016; Zhang et al. 2024; Lamb et al. 2020).

#### Novel Tank Diving Test (NTDT)

NTDT evaluates exploratory behavior by placing zebrafish in a new, unfamiliar tank. Zebrafish typically exhibits an initial "dive" to the bottom of the tank, which is followed by a period of exploration. The test measures how long zebrafish remain at the bottom or explore upper zones of the tank. Increased time spent at the bottom can indicate anxiety or stress-like behaviors, as zebrafish usually prefer to explore higher tank areas when not stressed. Methylmercury exposure, in particular, resulted in zebrafish spending more time at the lower part of the tank, showing signs of heightened anxiety due to potential neurobehavioral impairment (Hallauer et al. 2016; Haverroth et al. 2015; Wang et al. 2023; Lamb et al. 2020; Puty et al. 2014; Glazer et al. 2016).

#### Diving Tank Test (DTT)

Both DTT and the NTDT assess anxiety-like behaviors in zebrafish by placing them in unfamiliar environments, though they differ slightly in their approach. The DTT focuses on measuring how zebrafish respond to the stress of being placed in a new, confined space, usually by diving to the bottom and reducing exploration. The NTDT, on the other hand, typically involves observing zebrafish’s movement in a new tank, noting their time spent in different zones (e.g., top vs. bottom), with increased time spent in darker, lower areas indicating anxiety or stress. Both tests measure behavioral responses to environmental novelty, but the emphasis in DTT is on habituation and response to novelty, while NTDT focuses more on exploration patterns and space preference (Hallauer et al. 2016; Sackerman et al. 2010; Haverroth et al. 2015; Wang et al. 2023; Lamb et al. 2020; Puty et al. 2014; Glazer et al. 2016).

Thus, by analyzing changes in behavior following tumor induction or treatment, researchers can elucidate the underlying mechanisms that contribute to cognitive deficits associated with cancer (Table 3) (Clevenger et al. 2024; Shenoy et al. 2022).

**Table 3:** Behavioral and Social Effects of Various Substances on Zebrafish Following Different Exposure Conditions

Type of substance	Substance	Exposure dose	Exposure time	Type of test	Effects	Zebrafish	References
Probable human carcinogen (Group B2)	BaA	1 and 10 μM	Embryos at 8 hpf began BaA exposure until 4 dpf in 10-cm Petri dishes (∼30 embryos per dish)	LDT	Abnormal light–dark preference, with increased time in the dark, indicates heightened anxiety and sensitivity to stress	Embryos zebrafish	Zhang et al. 2024)
				SBT	Early social behavior impairments, with reduced interaction and response to social stimuli	Larvae and juvenile zebrafish	
				SBT	Social deficits were more severe, with reduced interaction and disrupted group dynamics, indicating long-term effects of embryonic exposure on neural pathways governing social behavior	Adult zebrafish	
				ST	Shoaling behavior was disrupted in BaA exposed zebrafish, leading to looser, less cohesive groups, reflecting social impairment and altered group coordination	Larvae zebrafish	
				OFT	Increased anxiety-like behavior was observed, with zebrafish spending more time near the edges (thigmotaxis) and less in the center, indicating anxiety and altered exploration	Larvae zebrafish	
Not classified as a carcinogen by the U.S. EPA	Atrazine	23.1825 nM–14.4891 μM	4 weeks	LDT	Increased time in the dark area suggests heightened anxiety and disrupted light/dark exploration, highlighting atrazine's anxiogenic effects on zebrafish	Adult zebrafish	Steinberg et al. 1995)
Possibly carcinogenic to humans (Group 2B)	Methylmercury	3.9 mM	24 h	LDT	A significant increase in time spent in the dark zone compared to controls indicates heightened anxiety-like responses in zebrafish, which typically explore light and dark zones more evenly. This increased preference for the dark zone suggests that methylmercury exposure elevates anxiety or stress-like behaviors, likely due to neurobehavioral impairment and potential disruptions in the serotonergic system	Adult zebrafish	Maximino et al. 2011; Puty et al. 2014)
				NTDT	Reduced exploration of the upper tank, spending more time in the lower portion, indicating anxiety-like behavior. Typically, less anxious zebrafish explore the upper areas, suggesting that methylmercury impairs exploratory behavior and increases anxiety, likely due to neurobehavioral deficits influenced by the serotonergic system		
Possible human carcinogens (Group 2A)	PCB	1260.3–1.2 nM	Developmental (4–24 hpf)	NTDT	Increased anxiety-like behavior and reduced exploration of upper areas, highlighting the lasting effects of early toxicant exposure on adult behavior	Adult zebrafish	Glazer et al. 2016)
Possibly carcinogenic to humans (Group 2B)	Nicotine	25 mg/L	30 min	DTT	Reduced anxiety-like behavior, spending more time in the upper areas of the tank. Additionally, variability in anxiety responses was observed across different genetic lines, highlighting the effects of both drugs and genetic influences on zebrafish behavior in novel environments	Adult zebrafish	Sackerman et al. 2010)
				LDT	Overt developmental toxicity, resulted in a lack of long- and short-term habituation to a novel environment in adults, as observed in the novel tank assay. This assay tests individual behavior in response to the stress of a new environment, where we typically expect an initial dive to the bottom of the tank followed by reduced exploration	Adult zebrafish	Sackerman et al. 2010)
Not classified as a carcinogen by IARC	CuSO_4_	0.015 mg/L	24 h	LDT	Altered light–dark preference. After exposure to copper, the zebrafish showed a significant increase in time spent in the dark zone, which is interpreted as an anxiety-like behavior	Adult zebrafish	Haverroth et al. 2015)
				NTDT	Signs of anxiety-like behavior. They spent more time at the bottom of the tank, a typical response in zebrafish experiencing stress or anxiety		
Known human carcinogen	As(III)	0, 50, 100, 300, and 500 ppb	72 hpf	SBT	Impaired social interactions, indicating social dysfunction as a consequence of embryonic exposure	Larvae zebrafish	Zhang et al. 2024)
		50, 100, and 300 ppb	chronic arsenite exposure (6 months)	NTDT	Decreased preference for the upper zone of the tank. They spent significantly more time in the lower, darker areas, which suggests an increase in anxiety-like behavior	Adult zebrafish	Hallauer et al. 2016)
				OFT	Reduced locomotor activity. They spent less time moving around and showed decreased exploratory behavior compared to control fish The study found that chronic arsenic exposure led to impaired exploration, further supporting the notion that it induced anxiety-like behaviors		
				SBT	They spent less time in proximity to conspecifics, which is a key aspect of normal social interaction in zebrafish		
		0, 100, 500 ppb	28 days	SBT	Reduced social interactions, spending less time near other fish compared to the control group	Larvae zebrafish	Wang et al. 2023)
				NTDT	Arsenic-exposed zebrafish spent more time in the bottom of the tank and less time exploring the upper areas, indicating heightened anxiety This behavior reinforces the idea that chronic exposure to inorganic arsenic induces anxiety-like symptoms in zebrafish		
Not classified as a carcinogen by the U.S. EPA	Atrazine	0 ppb 0.3 ppb, 3 ppb or 30 ppb	10 days	NTDT	Increased anxiety-like behavior. Specifically, these zebrafish spent significantly more time in the lower zone of the novel tank compared to control offspring, indicating a heightened level of anxiety	Larvae zebrafish	Lamb et al. 2020)
				OFT	Reduced locomotor activity. They were less active and spent more time in the corners of the arena, which is typically associated with avoidance behavior and increased anxiety		
				SBT	Reduced social interactions. They spent less time interacting with conspecifics compared to the control group. This suggests that the paternal exposure to the herbicide adversely affected their social dynamics and communication		

*BaA* benzo[a]anthracene, *CuSO*_*4*_ cooper sulfate, *dpf* days post-fertilization, *DTT* diving tank test, *hpf* hours post-fertilization, *IARC* International Agency for Research on Cancer, *LDT* light/dark test, *NTDT* novel tank diving Test, *OFT* open field test, *PCB* polychlorinated biphenyls, *SBT* social behavior test, *ST* shoaling test, *hpf* hours post-fertilization, *U.S. EPA* United States Environmental Protection Agency

On the other hand, behavioral assays allow for the assessment of therapeutic interventions aimed at mitigating cognitive decline in cancer-affected zebrafish, thereby providing insights that could translate into clinical applications for humans (Tan et al. 2022).

In Table 3, we present the behavioral tests of zebrafish exposed to both carcinogenic and non-carcinogenic substances.

The integration of behavioral tests in zebrafish models presents a robust framework for studying the effects of cancer on cognitive functions. By leveraging their unique biological attributes and advanced testing methodologies, researchers can gain valuable insights into the interplay between cancer and cognition. This approach not only enhances our understanding of disease mechanisms but also aids in the development of novel therapeutic strategies aimed at preserving cognitive health in cancer patients.

**Fig. 4:**
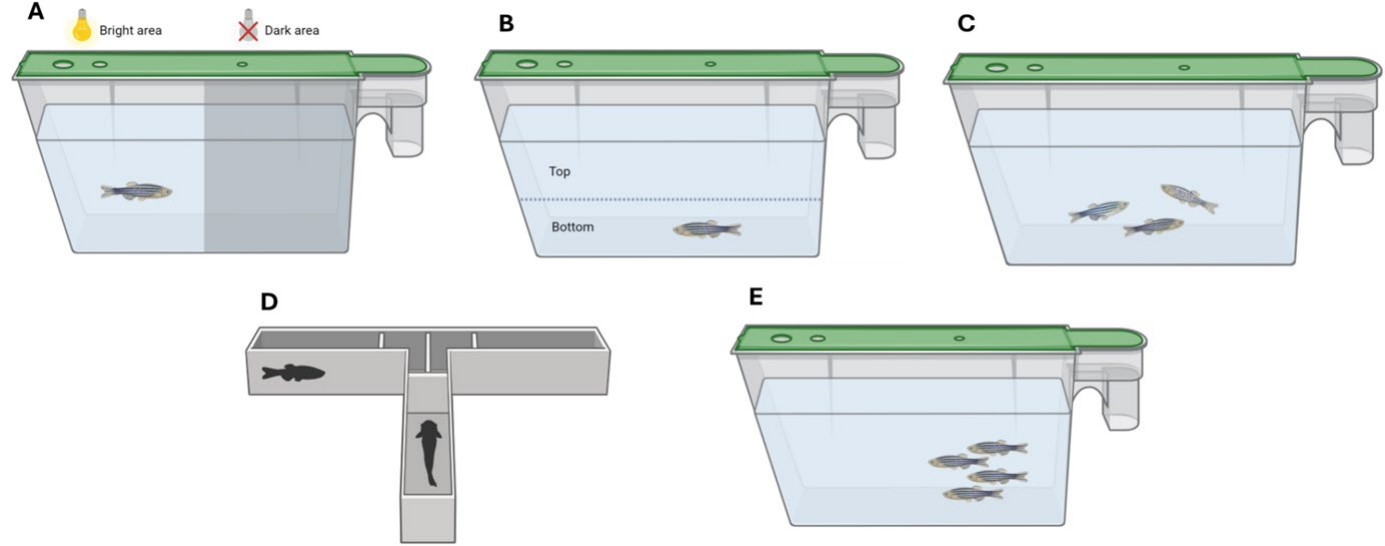
Schematic representation of the different assays used to assess various behavioral responses: **A** LDT (Light–Dark Test). **B** NTDT (Novel Tank Diving Test). **C** ST (Shoaling Test). **D** SBT (Social Behavior Test). **E** OFT (Open Field Test). These tests are designed to evaluate anxiety-like behaviors, locomotor activity, and social interactions in zebrafish model (Created in BioRender. Ionescu, C. (2025) https://BioRender.com/w37r782)

Below, we assessed an illustration of the behavioral tests conducted on zebrafish (Fig. 4).

## Limitations of Zebrafish as a Model in Cancer Research

Zebrafish have become an essential tool in cancer research due to their genetic similarities with humans, transparent embryos, and the ability to observe tumor growth in real time. However, while they offer unique advantages, there are several limitations that researchers must consider:

### Differences in Physiology

Although zebrafish share around 70% of human genes, their physiology differs significantly in areas such as immune system composition, metabolism, and hormonal regulation. These differences can affect how cancers develop and respond to treatments (Weiss et al. 2022; Chen et al. 2021).

### Simpler Tumor Microenvironment

Zebrafish do not replicate the complexity of the human tumor microenvironment. Elements such as stromal interactions, immune cell infiltration, and extracellular matrix remodeling—critical for tumor growth and metastasis—are less pronounced in zebrafish (Weiss et al. 2022; Chen et al. 2021).

### Temperature Constraints

As ectothermic organisms, zebrafish depend on external temperatures, typically maintained at 28–29 °C during experiments. This differs from the human body temperature of 37 °C and can influence tumor progression and drug metabolism, potentially affecting the translatability of results (Chen et al. 2021; Kirchberger et al. 2017).

### Differences in Organ Development and Function

Zebrafish organs, while analogous to human ones, are simpler in structure and function. For example, their liver and pancreas lack the cellular complexity found in humans, and their hematopoietic system operates differently, limiting their application in certain studies (Kirchberger et al. 2017).

### Scale and Size

Zebrafish are ideal for high-throughput studies due to their small size, but this can make it challenging to study large tumor masses or metastatic progression typical of advanced human cancers. Larger models like mice may be required for such studies (Weiss et al. 2022; Chen et al. 2021).

### Challenges with Long-Term Studies

While zebrafish are ideal for studying early stage cancer due to their rapid development, their relatively short lifespan poses a challenge for investigating long-term cancer progression and recurrence. This limitation makes it difficult to conduct studies that require prolonged observation or chronic treatment effects, restricting the ability to model long-term disease progression or metastasis in zebrafish (Caballero and Candiracci 2018; Vaz et al. 2019).

### Ethical and Translational Limitations

Although zebrafish are often preferred over mammalian models for ethical reasons, their findings still need to be validated in higher organisms, such as mice or larger mammals, to ensure their relevance to human treatments. These additional validation steps increase the complexity and time required to translate zebrafish-based discoveries into clinical applications (Caballero and Candiracci 2018; Vaz et al. 2019).

### Behavioral Testing in Zebrafish Models of Cancer: The Need for Complementary Biochemical and Histological Approaches

While behavioral tests in zebrafish, such as the light–dark test or open field test, provide valuable insights into anxiety-like and stress behaviors, they have limitations when it comes to studying cancer. These tests primarily focus on observable changes in behavior, which may not directly reflect underlying molecular or cellular alterations. To fully understand the impact of toxicants and carcinogens on zebrafish, additional biochemical and histological tests are necessary. These methods can help reveal more precise information about cellular damage, gene expression, and tissue changes that are indicative of cancer development and progression.

## Conclusions

This study highlights the critical role of zebrafish as an innovative and effective model organism in cancer research, particularly in the investigation of oxidative stress, genetic factors, and the effects of chemical exposures. The research emphasizes how various chemical agents, such as carcinogens, induce oxidative stress, DNA damage, and genetic mutations, which are key drivers of cancer development. Zebrafish provide a powerful platform for studying the genetic factors involved in tumor formation, including the roles of oncogenes (MYC, RAS, HER2) and tumor suppressor genes (TP53, RB1, BRCA1, BRCA2) offering insights into the molecular pathways that govern cancer initiation and progression.

In addition to genetic analysis, the study underscores the value of behavioral tests conducted on zebrafish exposed to carcinogenic chemicals. These tests enable the observation of neurological and cognitive effects that may arise as a result of chemical exposure, which can be indicative of underlying carcinogenic processes. By integrating behavioral assays such as the NTDT, LDT, DTT, OFT, SB, researchers can assess not only the physical and genetic impact of carcinogens but also the behavioral consequences that may mirror the cognitive and emotional disturbances seen in human cancer patients.

Overall, zebrafish serve as a multifaceted model for understanding the complex interactions between chemical exposures, genetic predisposition, and behavioral outcomes in cancer research. The ability to examine the molecular, genetic, and behavioral effects of carcinogens in a living organism offers unprecedented opportunities to explore the mechanistic pathways of cancer and evaluate potential therapeutic interventions. Overall, zebrafish prove to be an incredibly valuable tool for not only understanding the mechanisms behind cancer but also for discovering new treatments and therapies. The integration of 3D modeling, organ-on-chip, and tissue explant cultures with zebrafish can create complexity in preclinical cancer research and could revolutionize precision medicine studies. Future research should focus on optimizing these technologies to enhance the clinical relevance of preclinical models.

## Data Availability

No datasets were generated or analysed during the current study.
